# Matriptase zymogen supports epithelial development, homeostasis and regeneration

**DOI:** 10.1186/s12915-017-0384-4

**Published:** 2017-06-01

**Authors:** Stine Friis, Daniel Tadeo, Sylvain M. Le-Gall, Henrik Jessen Jürgensen, Katiuchia Uzzun Sales, Eric Camerer, Thomas H. Bugge

**Affiliations:** 1 Proteases and Tissue Remodeling Section, Oral and Pharyngeal Cancer Branch, National Institute of Dental and Craniofacial Research, National Institutes of Health, 30 Convent Drive, Room 320, Bethesda, MD 20892 USA; 2 Section for Molecular Disease Biology, Department of Veterinary Disease Biology, Faculty of Health and Medical Sciences, University of Copenhagen, Copenhagen, Denmark; 3 INSERM U970, Paris Cardiovascular Research Centre, Paris, France; 4 Université Sorbonne Paris Cité, Paris, France; 5 Department of Cell and Molecular Biology, Ribierão Preto School of Medicine, University of Sao Paulo, Sao Paulo, Brazil; 6 Georgetown University School of Medicine, Washington, DC, 20057 USA

**Keywords:** Development, Cell surface proteolysis, Epithelial cell signaling, Zymogen activity

## Abstract

**Background:**

Matriptase is a membrane serine protease essential for epithelial development, homeostasis, and regeneration, as well as a central orchestrator of pathogenic pericellular signaling in the context of inflammatory and proliferative diseases. Matriptase is an unusual protease in that its zymogen displays measurable enzymatic activity.

**Results:**

Here, we used gain and loss of function genetics to investigate the possible biological functions of zymogen matriptase. Unexpectedly, transgenic mice mis-expressing a zymogen-locked version of matriptase in the epidermis displayed pathologies previously reported for transgenic mice mis-expressing wildtype epidermal matriptase. Equally surprising, mice engineered to express only zymogen-locked endogenous matriptase, unlike matriptase null mice, were viable, developed epithelial barrier function, and regenerated the injured epithelium. Compatible with these observations, wildtype and zymogen-locked matriptase were equipotent activators of PAR-2 inflammatory signaling.

**Conclusion:**

The study demonstrates that the matriptase zymogen is biologically active and is capable of executing developmental and homeostatic functions of the protease.

**Electronic supplementary material:**

The online version of this article (doi:10.1186/s12915-017-0384-4) contains supplementary material, which is available to authorized users.

## Background

Trypsin-like serine proteases constitute a large family of proteolytic enzymes with diverse functions in vertebrate physiology [1, 2]. Common to members of this family is a conserved catalytic domain, which contains a Ser-Asp-His catalytic triad in the active site. All members of this protease family are synthesized as pro-enzymes (zymogens) with an N-terminal extension that spatially distorts the active site to render the protease catalytically inactive. Conversion to the active protease (zymogen conversion) occurs by removal of the N-terminal extension by a single proteolytic cleavage after an Arg or Lys residue, which is located within a highly conserved activation cleavage site. Removal of this N-terminal extension spatially reorients the catalytic domain to its active conformation [3, 4].

Matriptase is a membrane-anchored trypsin-like serine protease that is found in all vertebrates and is expressed in the epithelium of most tissues [5]. Previous studies have uncovered essential functions of matriptase in key aspects of epithelial biology, including epithelial development, tight junction formation, fluid secretion, and regeneration of damaged epithelium [6–13]. Deregulated matriptase activity has also been linked to growth factor and inflammatory signaling in the context of cancer and other diseases [14–26].

Matriptase is a modular, approximately 95-kDa type II transmembrane protein that consists of a cytoplasmic N-terminal domain, a signal anchor that serves as a single-pass transmembrane domain, a sea urchin sperm protein, enteropeptidase, agrin domain, two complement C1r/s urchin embryonic growth factor and bone morphogenetic protein-1 domains, four low-density lipoprotein receptor class A domains, and a C-terminal trypsin-like serine protease domain [21, 27, 28]. Zymogen conversion of matriptase occurs by hydrolysis of a specific Arg^614^-Val^615^ bond within the amino acid sequence RQAR^614^-VVGG [29–31]. Matriptase zymogen conversion on the surface of epithelial cells is followed by rapid inactivation of the protease by hepatocyte growth factor activator inhibitor (HAI)-1 and shedding of the protease-inhibitor complex from the cell surface [32, 33].

Matriptase is unusual among trypsin-like serine proteases in that the zymogen possesses measurable enzymatic activity. This was first evidenced by the ability of the purified soluble matriptase serine protease domain to undergo auto-activation when incubated for prolonged periods [34]. Subsequent studies showed that the ability of full-length matriptase to undergo activation site cleavage in cell-based assays was dependent on the integrity of the matriptase catalytic triad [30, 35]. A careful biochemical analysis of a soluble zymogen-locked version of matriptase comprising the entire ectodomain of the protease showed that the zymogen displayed 30-fold lower activity than the activated form of the enzyme towards a small peptide substrate that closely resembles the matriptase activation cleavage site sequence [36]. This finding led the authors to propose that the principal function of the enzymatic activity of the matriptase zymogen is to facilitate matriptase zymogen conversion. On the other hand, using a cell-based assay, we have recently demonstrated that several zymogen-locked matriptase mutants, but not a catalytically inactive matriptase mutant, are capable of activating prostasin – a validated matriptase substrate [37]. These findings give rise to two hypotheses, namely that (1) the intrinsic activity of the matriptase zymogen serves exclusively to mediate matriptase zymogen conversion and that (2) the matriptase zymogen is a biologically active molecule capable of cleaving heterologous proteins to execute essential biological functions of the protein.

In this study, we tested these hypotheses by generating transgenic mice expressing matriptase active site and activation cleavage site mutants, and by editing the endogenous matriptase gene (*St14*). We show that epidermal mis-expression of a zymogen-locked, but not a catalytically inactive, matriptase transgene causes skin pathology similar to that previously observed in transgenic mice expressing wildtype matriptase [14]. Furthermore, we show that, unlike matriptase null mice, mice engineered to express only zymogen-locked endogenous matriptase complete development have normal postnatal survival, develop epithelial barrier function, and are capable of regenerating damaged epithelium.

## Results

### Zymogen-locked matriptase is biologically active when expressed in basal keratinocytes of transgenic mice

**Fig. 1:**
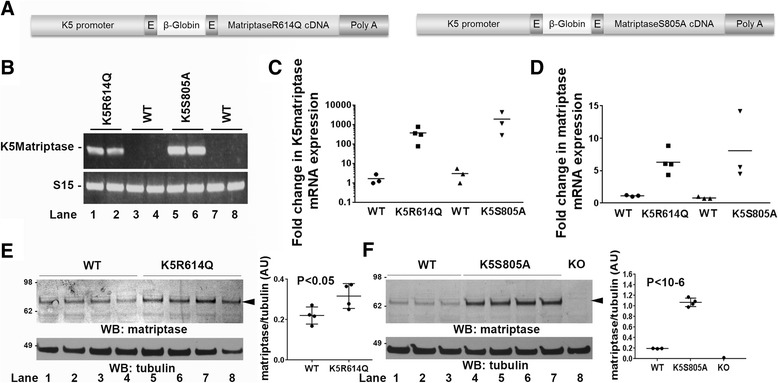
Generation of keratin-5-matriptase zymogen-locked and keratin-5-matriptase catalytically inactive transgenic mice. **a** Schematic structure of the keratin-5-matriptase transgenes. The bovine keratin-5 promoter (K5), rabbit β-globin exons, rabbit β-globin intron B, mouse matriptase cDNA, and rabbit β-globin polyadenylation signal (Poly A) are shown. **b** Expression of the keratin-5-matriptase transgenes in mouse skin. RT-PCR analysis of skin from an established keratin-5-matriptase zymogen (K5R614Q, lanes 1 and 2) transgenic mouse line, two wildtype littermates (WT, lanes 3 and 4), an established keratin-5-matriptase catalytically inactive (K5S805A, lanes 5 and 6) transgenic mouse line, and two wildtype littermates (WT, lanes 7 and 8). **c** and **d** Quantitative real-time PCR analysis with primer pairs specific for transgenic matriptase mRNA (**c**) or total matriptase mRNA (**d**) in skin from newborn wildtype mice (WT, n = 3 mice), keratin-5-matriptase zymogen-locked transgenic (K5R614Q, n = 4 mice) littermates, wildtype mice (WT, n = 3 mice), and keratin-5-matriptase catalytically inactive transgenic (K5S805A, n = 3 mice) littermates. *Horizontal bars* are means of individual values. **e** Western blot analysis of total matriptase protein in skin from newborn wildtype mice (lanes 1–4) and keratin-5-matriptase zymogen-locked (K5R614Q, lanes 5–8) littermates. Quantification of band intensity from scanned Western blot from (e) is shown on the *right* (mean ± SD). **f** Western blot analysis of matriptase in skin from newborn wildtype mice (lanes 1–3) and keratin-5-matriptase catalytically inactive (K5S805A, lanes 4–7) littermates. Matriptase null skin was included as negative control (KO, lane 8). Molecular weight markers are shown on the *left*. Quantification of band intensity from scanned Western blot from F is shown on the *right* (mean ± SD). *P* values were determined by Student’s *t* test. Additional file 1: Raw supporting data

We have previously shown that mis-expression of wildtype matriptase in the basal keratinocyte compartment of transgenic mice causes alopecia and progressive interfollicular hyperplasia through the dual activation of c-met and proteinase activated receptor (PAR)-2 signaling [14, 18, 19]. We used this observation to determine if zymogen-locked matriptase displays biological activity in an in vivo setting. Specifically, we generated transgenic mice expressing zymogen-locked matriptase by introducing a R614Q point mutation in the activation cleavage site, which prevents activation site cleavage required for formation of the substrate binding pocket and alignment of the serine-histidine-aspartic acid triad [4]. We also generated transgenic mice expressing catalytically inactive matriptase by introducing a S805A mutation, which replaces the serine of the catalytic serine-histidine-aspartic acid triad. Both matriptase mutants were expressed under the control of a keratin-5 promoter (Fig. 1a), which targets expression of linked genes to basal keratinocytes of the interfollicular epidermis and outer root sheath of hair follicles. Four founders harboring the K5R614Q transgene were obtained by pronuclear injection of zygotes. One founder displayed severe alopecia at the time of weaning and did not transmit the transgene. Two founders had noticeable alopecia at the time of weaning, and one founder had no noticeable outward phenotype and a phenotype was also not observed in the F1 offspring. Four founders harboring the K5S805A transgene were obtained, of which one was nulliparous. The remaining three founders transmitted the transgene to the F1 generation. No outwardly observable phenotype was found in any of the four founders or in the F1 offspring.

**Fig. 2:**
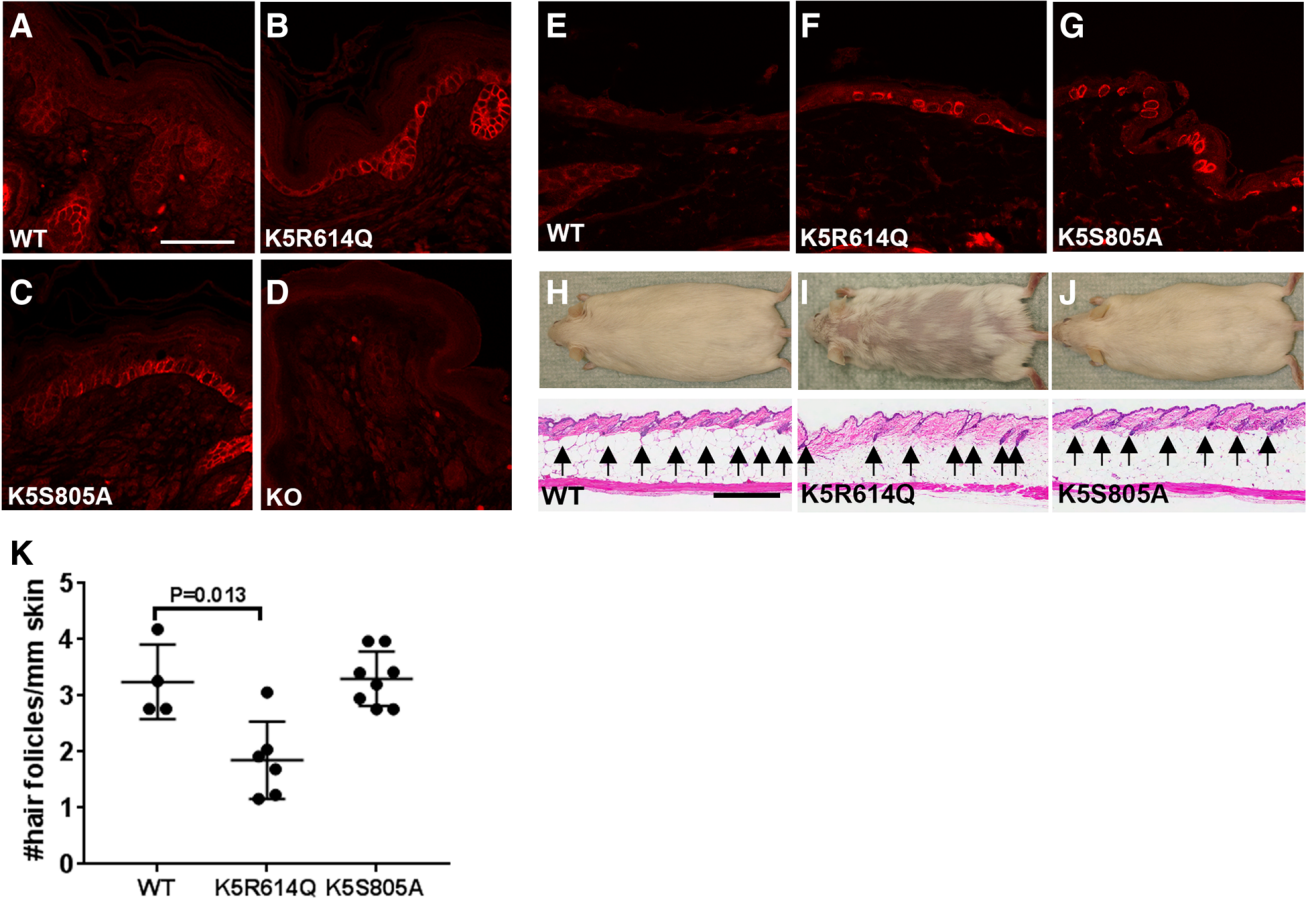
Zymogen-locked matriptase is biologically active when expressed in basal keratinocytes of transgenic mice. Matriptase protein expression in the skin of newborn wildtype mice (WT) (**a**), keratin-5-matriptase zymogen-locked transgenic mice (**b**), keratin-5-matriptase catalytically inactive mice (**c**), matriptase null mice (**d**), adult wildtype mice (**e**), keratin-5-matriptase zymogen-locked transgenic mice (**f**), and keratin-5-matriptase catalytically inactive transgenic mice (**g**). Scale bar = 50 μm, representative for **a**–**g. h–j** Outward appearance (*top panels*) and histological appearance (*bottom panels*, **h** and **e**) of 1-year-old wildtype (**h**), keratin-5-matriptase zymogen-locked (**i**), and keratin-5-matriptase catalytically inactive (**j**) mice. Scale bar = 500 μm, representative for **h**–**j. k** Quantification of number of hair follicles/mm in full skin sections stained with hematoxylin and eosin from wildtype (WT, n = 4 mice), keratin-5-matriptase zymogen-locked transgenic (K5R614Q, n = 6 mice), and keratin-5-matriptase catalytically inactive transgenic (K5S805A, n = 7 mice) mice (mean ± SD). Expression of zymogen-locked matriptase, but not catalytically inactive matriptase in basal keratinocytes causes alopecia (*top panels*) associated with reduced hair follicle density. Additional file 1: Raw supporting data

Detailed characterization of one established line harboring the R614Q matriptase transgene (K5R614Q) and one established line harboring the S805A matriptase transgene (K5S805A) showed that they expressed mRNA for each of these transgenes in the skin (Fig. 1b, c), which resulted in a comparable increase in total skin matriptase mRNA (Fig. 1d). This increase in matriptase mRNA resulted in a modest (approximately 1.5-fold) increase in total skin matriptase protein in the K5R614Q transgenic line (Fig. 1e) and in an approximately five-fold increase in total skin matriptase protein in the K5S805A transgenic line (Fig. 1f). As expected, immunofluorescence staining of skin from newborn and adult mice showed expression of matriptase in basal keratinocytes of both transgenic lines, which was not detectable in wildtype littermate skin (compare Fig. 2a with b and c, and e with f and g). The specificity of the staining was demonstrated by the absence of staining of matriptase-deficient skin (Fig. 2d). The two established K5R614Q and K5S805A transgenic lines were indistinguishable from wildtype mice at birth and for the first 3–6 weeks of life (data not shown). However, the K5R614Q mice developed alopecia of the dorsal and facial skin with a patchy appearance, resulting in areas of skin largely devoid of pelage hair within 6–12 months (compare Fig. 2h and i, top panels). The progressive alopecia of K5R614Q mice was associated with markedly fewer hair follicles (compare Fig. 2h and i, bottom panels, quantified in k), as also observed in transgenic mice expressing wildtype matriptase [14]. However, interfollicular hyperplasia, a phenotype associated with transgenic expression of wildtype matriptase, was not observed. In contrast, no phenotype was apparent in K5S805A mice, even when followed for up to 1 year (Fig. 2j), despite the identical expression pattern and higher level of expression of S805A matriptase protein. Taken together, this shows that zymogen-locked matriptase is endowed with biological activity in vivo, that it can elicit one of the phenotypes (progressive alopecia) elicited by wildtype matriptase when mis-expressed in mouse epidermis, and that the biological activity of zymogen matriptase likely depends on the integrity of the catalytic triad.

### Engineering mice expressing zymogen-locked endogenous matriptase

**Fig. 3:**
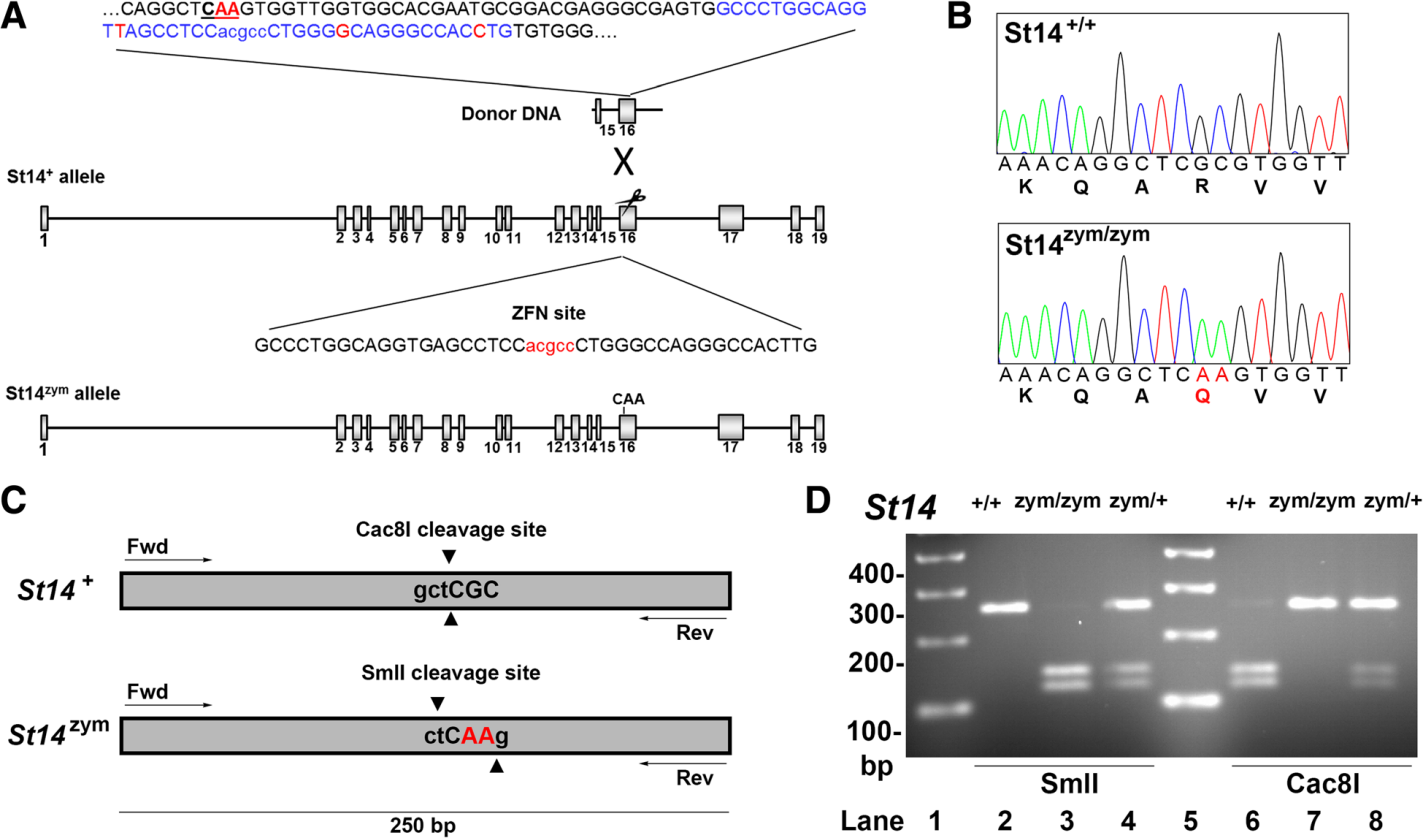
Generation of mice expressing zymogen-locked endogenous matriptase by ZFN-mediated gene editing. **a** Structure of donor DNA (*top*), wildtype *St14* allele with the ZFN binding site (*middle*), and edited *St14* allele (*bottom*) containing the base pair substitutions of interest. Exons are indicated as *gray boxes* and introns as *black lines*. The highlighted part of the donor DNA shows the dinucleotide substitution (indicated in *red letters* and *underlined*), the ZFN binding site (*blue letters*), and the synonymous nucleotide changes of the donor DNA (*red letters*) to avoid ZFN cleavage of the donor DNA. The ZFN binding site is shown below in *capital letters* with the ZFN cleavage site in *red*. Introduction of the donor DNA at the ZFN target site results in a c.1841–1842 GC → AA dinucleotide substitution. **b** Sequence analysis of exon 16 from *St14* ^+/+^ (*top panel*) and *St14* ^zym/zym^ (*bottom panel*) mice confirms the GC → AA dinucleotide substitution causing the arginine 614 to glutamine substitution in endogenous matriptase (indicated by *red letters* in nucleotide and amino acid sequence). **c** and **d** Genotyping of *St14* ^zym/zym^ mice. **c** The GC → AA dinucleotide substitution in endogenous *St14* gene causes the loss of a Cac8I restriction endonuclease cleavage site and the generation of a SmlI restriction endonuclease cleavage site. **d** PCR amplification of genomic DNA from the targeted *St14* exon 14 region from *St14* ^+/+^ (lanes 2 and 6), *St14* ^zym/zym^ (lanes 3 and 7), and *St14* ^zym/+^ (lanes 4 and 8) mice. The amplified DNA was digested with SmlI in lanes 2–4 and with Cac8I in lanes 6–8. Position of molecular weight markers (lanes 1 and 5) are indicated on the *left* (bp)

**Fig. 4:**
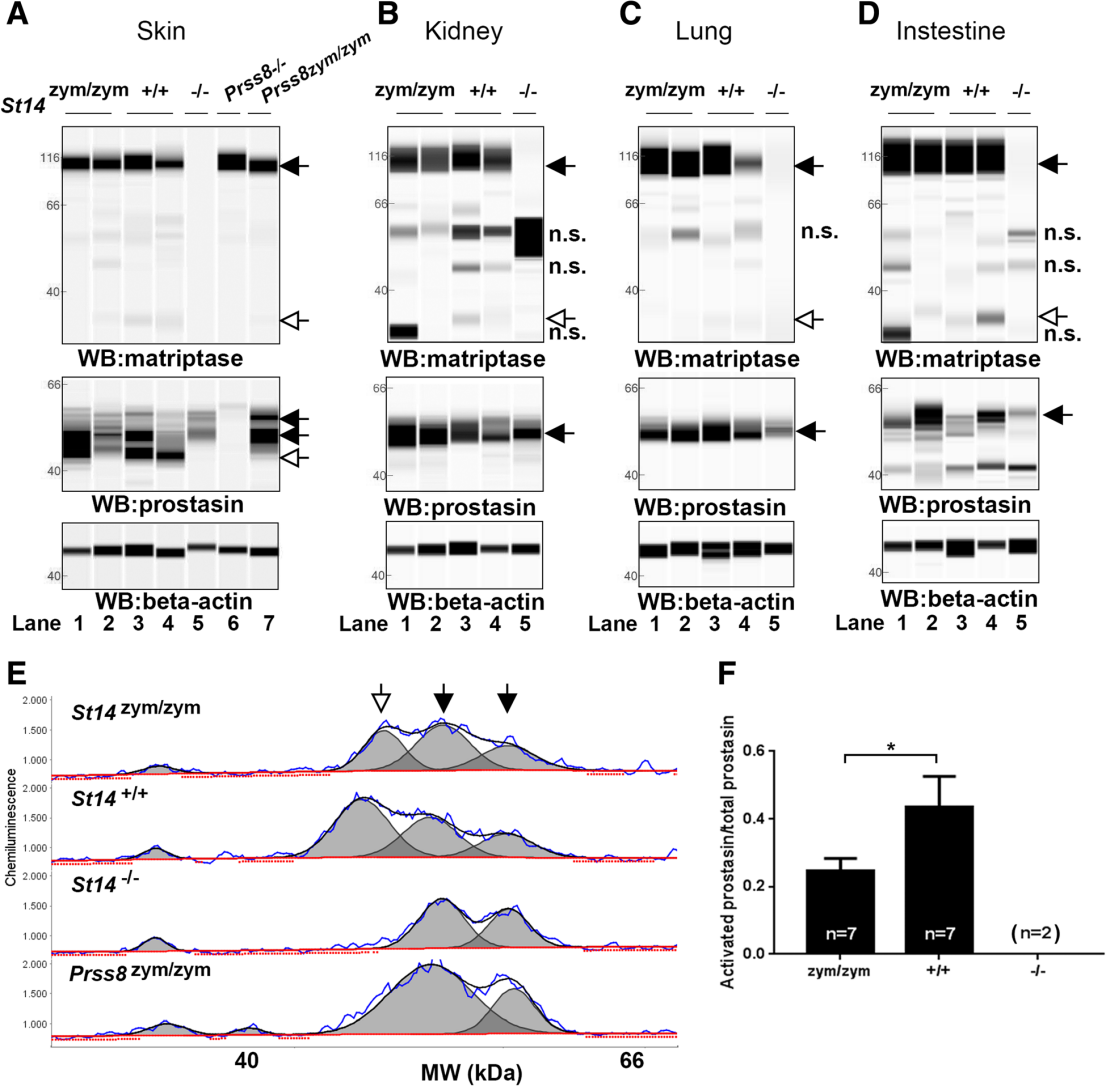
Zymogen-locked matriptase induces epidermal prostasin processing. Protein extracts from skin (**a**), kidney (**b**), lung (**c**), and intestine (**d**) from newborn *St14* ^zym/zym^ (lanes 1 and 2), *St14* ^+/+^ (lanes 3 and 4), and *St14* ^*–/–*^ (lane 5) littermates were separated by capillary electrophoresis and probed with antibodies against matriptase (*top panels*), prostasin (*middle panels*), or β-actin (*bottom panels*). Lanes 6 and 7 in (a) are skin extracts from prostasin null (*Prss8* ^*–/–*^) and prostasin zymogen-locked (*Prss8* ^*zym/zym*^) mice, respectively. Zymogens of matriptase and prostasin are indicated with *filled arrows*, and the activated forms are indicated with *open arrows*. n.s. non-specific. Positions of molecular weight markers (kDa) are indicated on the left. **e** Representative example of quantification of activated prostasin (*open arrow*) and zymogen prostasin (*filled arrows*) in protein extracts from skin from a newborn *St14* ^zym/zym^ mouse (top panel), *St14* ^*+/+*^ mouse (*second panel from top*), and a newborn *St14* ^–/–^ mouse (*second panel from bottom*). Skin extracts from a newborn mouse expressing zymogen-locked (*Prss8* ^zym/zym^) endogenous prostasin is included as reference (bottom panel). **f** Ratio of activated prostasin to total prostasin in skin extracts from newborn *St14* ^zym/zym^ (*left bar*, n = 7), *St14* ^+/+^ (*middle bar*, n = 7), and *St14* ^*–/–*^ (*right bar*, n = 2) mice, quantified as in (**e**). Data are shown as mean ± SD. *P = 0.0011 was determined by one-way ANOVA, two-tailed. Additional file 1: Raw supporting data

To determine if zymogen-locked matriptase could not only mediate pathological functions of the protease but also perform key physiological functions, we next generated mice expressing only zymogen-locked endogenous matriptase. Specifically, we introduced a c.1841–1842 GC → AA di-nucleotide substitution into exon 16 of the *St14* gene by template-guided repair of a zinc finger nuclease (ZFN)-induced double strand DNA break in FVB/NJ zygotes (Fig. 3a). This dinucleotide change generated a mutant *St14* allele (hereafter referred to as *St14* ^*zym*^) that encodes a matriptase with Arg614 substituted by Gln, thus rendering the mutant matriptase refractory to activation site cleavage, as shown previously [37]. Introduction of the dinucleotide change was verified by sequencing analysis of DNA from mice bred to homozygosity for the mutated allele (Fig. 3b). The two base pair substitutions from GC to AA resulted in the elimination of a Cac8I restriction endonuclease cleavage site and the generation of a SmlI restriction endonuclease cleavage site, which allowed for convenient genotyping of mice by PCR amplification of the mutated region, followed by digestion of the amplified DNA by either Cac8I or SmlI (Fig. 3c, d). For detection of matriptase protein, we used a polyclonal antibody raised against human matriptase, which cross reacts with mouse matriptase and has been used previously to detect the protease in intact mouse tissues and in tissue lysates [38]. This antibody recognizes the matriptase serine protease domain, allowing for identification of activated matriptase and matriptase zymogen by SDS/PAGE under reducing conditions. Preliminary analysis showed that detection of the serine protease domain of activation site-cleaved matriptase in mouse tissues using this antibody by standard SDS/PAGE followed by Western blot was difficult due to the sensitivity of the antibody. Likewise, because prostasin is heterogeneously glycosylated and undergoes C-terminal processing, and because activation site cleavage and reduction of prostasin leads to the removal of just 12 amino acids [39], analysis of the state of activation of prostasin proved difficult using standard techniques. We therefore employed the Peggy Sue capillary electrophoresis system, which separates proteins by size with high resolution and detects them by immunoassay in a sensitive and quantitative format [40], to examine the expression of matriptase and prostasin in the gene-edited mice. To aid the identification of the various forms of prostasin, we included in parallel skin from mice expressing zymogen-locked endogenous prostasin [41]. Analysis of protein extracts from skin, kidney, lungs, and intestine of newborn *St14* ^zym/zym^ mice and wildtype (*St14* ^+/+^) littermates showed that the mutant matriptase was expressed at levels similar to wildtype matriptase (Fig. 4a–d, top panels, black arrow, compare lanes 1 and 2 with 3 and 4). As expected, whereas matriptase from *St14* ^+/+^ mice presented in these tissues as a dominant 95 kDa band corresponding to zymogen matriptase, and a much less abundant 30 kDa band corresponding to activated matriptase [42], only the matriptase zymogen was found in tissues from *St14* ^zym/zym^ mice (Fig. 4a–d, top panels, white arrow, compare lanes 1 and 2 with lanes 3 and 4). Antibody specificity was demonstrated by the absence of these two immunoreactive proteins in the same tissues from *St14* ^*–/–*^ mice analyzed in parallel (Fig. 4a–d, lanes 5). The same protein extracts were next analyzed for the expression of prostasin (Fig. 4a–d, middle panels). Interestingly, prostasin was found in its activation site-cleaved form in the epidermis of both *St14* ^zym/zym^ mice and *St14* ^+/+^ mice (Fig. 4a, middle panel, left, white arrow, compare lanes 1–2 to lanes 3–4), but not in *St14* ^*–/–*^ mice (lanes 5), as previously shown [42]. Skin lysates from prostasin null mice (*Prss8* ^*–/–*^) and prostasin zymogen-locked mice (*Prss8* ^zym/zym^) were used as controls (Fig. 4a, middle panel, lanes 6 and 7, respectively). Quantitative analysis of skin extracts (Fig. 4e, f) showed that the ratio of activated prostasin to prostasin zymogen was reduced by 48% in newborn *St14* ^zym/zym^ mice as compared to *St14* ^+/+^ mice, whereas active prostasin was undetectable in newborn *St14* ^*–/–*^ epidermis. A similar analysis of kidney, lung, and intestine was inconclusive as to the ratio of activated prostasin to prostasin zymogen in these tissues due to insufficient chromatographic separation of the various immunoreactive protein species (Fig. 4b–d, middle panels and data not shown).

### Zymogen-locked matriptase supports epithelial development, homeostasis, and regeneration

**Fig. 5:**
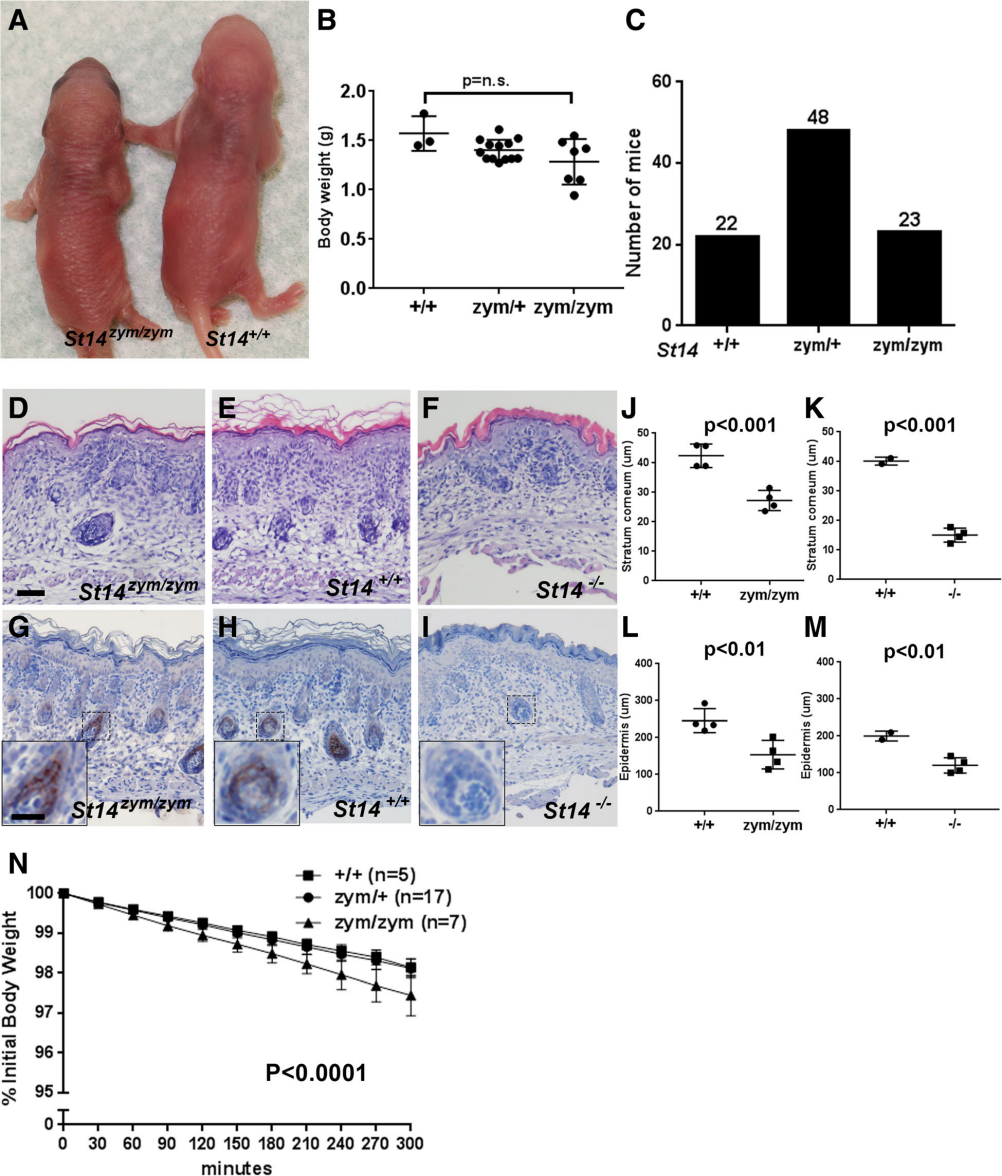
Zymogen-locked endogenous matriptase supports interfollicular epidermal development and epidermal barrier acquisition. **a** Outward appearance of newborn *St14* ^zym/zym^ (*left*) and *St14* ^+/+^ (*right*) pups. **b** Body weights of newborn *St14* ^+/+^ (*left bar*, n = 3), *St14* ^zym/+^ (*middle bar*, n = 13), and *St14* ^zym/+^ (*right bar*, n = 7) pups. Data are shown as mean ± SD. *P* = non-significant. One-way ANOVA, two-tailed. **c** Normal postnatal survival of mice expressing zymogen-locked endogenous matriptase. Genotype distribution of offspring from interbred *St14* ^zym/+^ mice. Genotypes were obtained at weaning from 93 offspring from 14 litters. Hematoxylin and eosin staining (**d–f**) and matriptase immunohistochemistry (**g–i**) of skin from newborn *St14* ^zym/zym^ pups (**d** and **g**) and wildtype littermates (**e** and **h**). Skin from newborn *St14* ^–/–^ mice was included as comparison for stratum corneum morphology (**f**) and as control for antibody specificity (**i**). Scale bar in d = 50 μm, representative for **d–i**. Scale bar in inset in g = 20 μm, representative for inset in g–i. Quantification of stratum corneum thickness of newborn epidermis of *St14* ^+/+^ pups (left bar, n = 4) and *St14* ^zym/zym^ littermates (*right bar*, n = 4) (**j**), and of newborn epidermis of *St14* ^+/+^ pups (*left bar*, n = 2) and *St14* ^–/–^ littermates (*right bar*, n = 4) (**k**). Data are shown as mean ± SD. Quantification of epidermal thickness of newborn epidermis of *St14* ^+/+^ pups (*left bar*, n = 4) and *St14* ^zym/zym^ littermates (*right bar*, n = 4) (**l**), and of newborn epidermis of St14^+/+^ pups (*left bar*, n = 2) and *St14* ^–/–^ littermates (*right bar*, n = 4) (**m**). Data are shown as mean ± SD. *P* values were determined by Student’s *t* test, two-tailed. **n** Zymogen-locked matriptase supports epidermal barrier formation. Rate of epidermal fluid loss from newborn mice was estimated by measuring reduction of body weight as a function of time. The data are expressed as the average percentage of initial body weight for *St14* ^+/+^ pups (squares; n = 5), *St14* ^zym/+^ pups (circles; n = 17), and *St14* ^zym/zym^ (triangles; n = 7) littermates (mean ± SD). Error bars indicate SD, *P* < 0.0001 (linear regression analysis, two-tailed). Additional file 1: Raw supporting data

Congenital or conditional matriptase deficiency causes embryonic or postnatal lethality in mice due to the catastrophic loss of barrier function of both simple and multi-layered epithelium [6, 7, 9, 11, 43]. To determine if zymogen-locked matriptase can support epithelial development and homeostasis, we next interbred *St14* ^+/zym^ mice and genotyped 93 offspring from a total of 14 litters. Surprisingly, the epidermis of newborn *St14* ^zym/zym^ pups was outwardly indistinguishable from *St14* ^+/+^ littermates (Fig. 5a), and the body weight of newborn *St14* ^zym/zym^ pups was not significantly different from their *St14* ^+/zym^ and *St14* littermates (Fig. 5b). Furthermore, *St14* ^zym/zym^ pups were present at weaning at the expected Mendelian frequency (Fig. 5c). These unexpected findings indicate that matriptase can support postnatal development independent of zymogen conversion. Newborn *St14* ^zym/zym^ skin presented histologically with a mildly compacted, slightly more immature stratum corneum (compare Fig. 5d and e, quantified in j), and was different from the immature compacted stratum corneum of *St14* ^–/–^ pups (compare Fig. 5d and f, quantified in k). Epidermal thickness (excluding stratum corneum) was reduced in both newborn *St14* ^zym/zym^ epidermis and *St14* ^–/–^ epidermis (quantified in Fig. 5l and m). The expression pattern of zymogen matriptase in skin of newborn mice was indistinguishable from that of wildtype littermates (Fig. 5, compare g and h). Note, however, that antibody sensitivity allowed for detection of matriptase in hair follicles, but not for detection of interfollicular suprabasal matriptase documented in previous studies [16, 44]. Skin from matriptase null mice was included as a negative control (Fig. 5i). Direct analysis of transepidermal fluid loss rates revealed only a minimal increase in newborn *St14* ^zym/zym^ pups (0.51% of body weight per hour), as compared to *St14* ^zym/+^ (0.37% of body weight per hour) and *St14* ^+/+^ littermates (0.36% of body weight per hour) (Fig. 5n), which was much lower than the fluid loss rates previously observed in *St14* ^–/–^ pups (2.09% of body weight per hour) [6, 7].

**Fig. 6:**
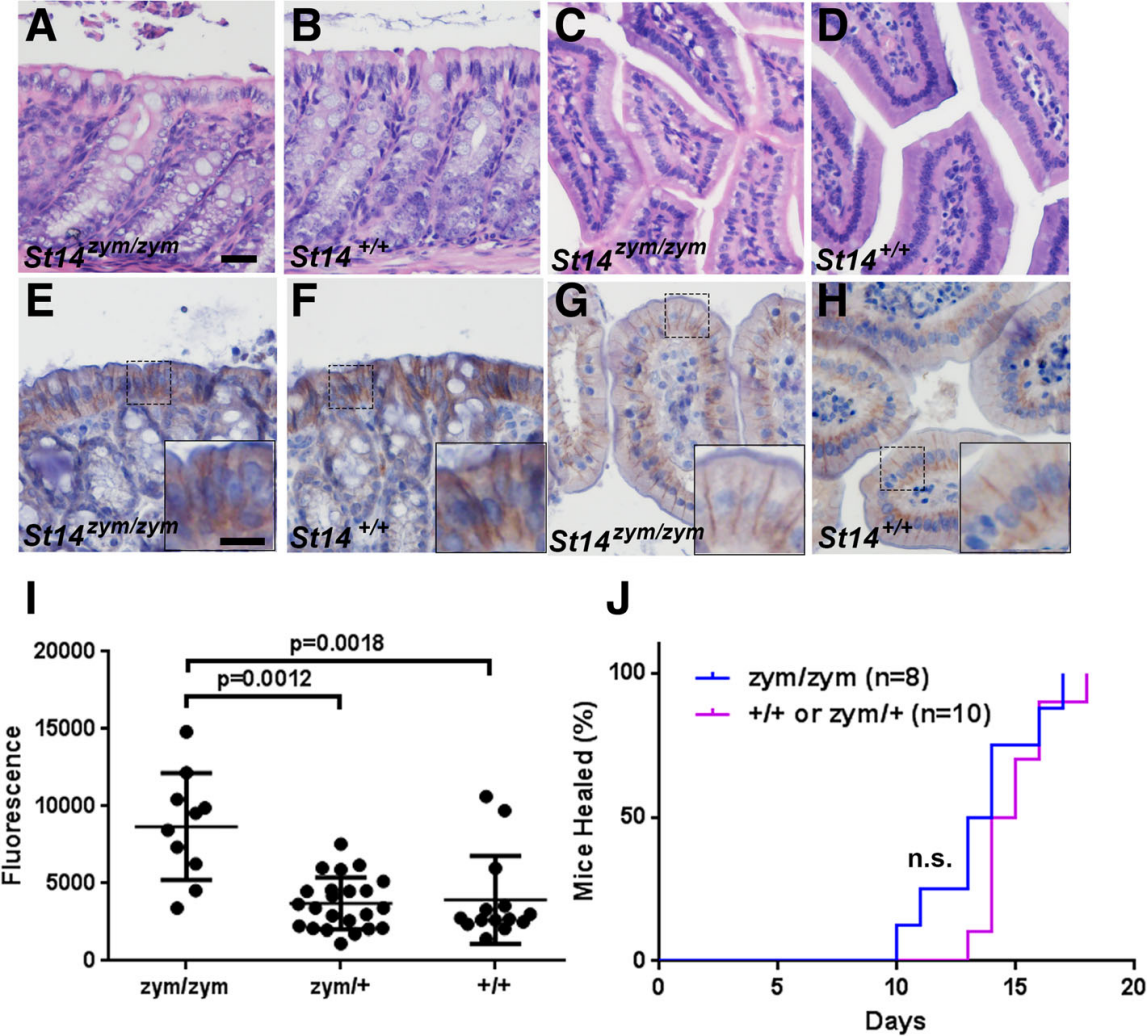
Zymogen-locked endogenous matriptase supports intestinal barrier formation and epidermal regeneration. Histological appearance (**a–e, h**) and matriptase immunohistochemistry (**e–h**) of large (**a**, **b**, **e**, **f**) and small (**c**, **d**, **g**, **h**) intestine of 3-month-old *St14* ^zym/zym^ mice (**a**, **c**, **e**, **g**) or wildtype littermates (**b**, **d**, **f**, **h**). Scale bar in (**a**) = 20 μm, representative for **a–h**. Scale bar in inset in (e) = 10 μm, representative for insets in **e–h. i** Concentration of FITC-labeled dextran in the blood of 4- to 8-week-old *St14* ^*zym/zym*^ mice (n = 10), *St14* ^*zym/+*^ mice (n = 23), and *St14* ^*+/+*^ littermates (n = 14) 3 h after instillation of FITC-labeled dextran into the stomach by oral gavage. A small, but significant, increase in intestinal permeability was observed in *St14* ^*zym/zym*^ mice as compared to littermate *St14* ^*+*^ mice. One-way ANOVA, non-parametric, two tailed. **j** Rate of healing of 1.5-cm incisional skin wounds in *St14* ^+^ (*red*, n = 10) mice and *St14* ^*zym/zym*^ (*blue*, n = 8) littermates. Wound healing was not significantly delayed in *St14* ^*zym/zym*^ mice relative to *St14* ^*+*^ littermates. Additional file 1: Raw supporting data

Congenital or conditional deletion of matriptase from intestinal epithelial cells of mice causes a catastrophic increase in intestinal barrier permeability, gross disruption of intestinal tissue architecture, failure to thrive, and high mortality [9]. In sharp contrast, intestinal tissues from adult *St14* ^zym/zym^ mice were histologically unremarkable (Fig. 6, compare a to b and c to d). The expression pattern of zymogen matriptase in the epithelial cells of the intestines was indistinguishable from that of wildtype littermates (Fig. 6, compare e to f and g to h). Direct measurement of barrier permeability in adult *St14* ^zym/zym^ intestine, as determined by the rate of gavaged FITC-dextran entering the blood stream (Fig. 6i), was approximately doubled, as compared to *St14* ^zym/+^ and *St14* ^+/+^ littermates (median 8600 fluorescence units, as opposed to 3700 fluorescence units and 3900 fluorescence units, respectively), which is a much lower rate than the previously reported 10-fold increase in barrier permeability of *St14* ^–/–^ intestine [9]. This finding shows that zymogen-locked matriptase can partially fulfill the role of wildtype matriptase in intestinal barrier homeostasis.

We recently reported that normal skin wound healing requires prostasin proteolytic activity, as evidenced by delayed wound healing in mice expressing catalytically inactive prostasin [45]. To determine if zymogen-locked matriptase could support this prostasin-dependent repair process, we next generated 1.5-cm full-thickness incisional skin wounds in the mid-scapular dorsal region of *St14* ^*zym/zym*^ mice and their *St14* ^zym/+^ and *St14* ^+/+^ littermates. The wounds were left unsutured and undressed and were observed daily by an investigator blinded as to mouse genotype. The wounds were scored as healed based on the macroscopic closure of the incision interface and restoration of epithelial covering. Interestingly, mice expressing zymogen-locked matriptase were capable of healing their wounds within the same time frame as their wildtype littermates (Fig. 6j).

### Abnormal hair follicle development in mice expressing zymogen-locked endogenous matriptase

**Fig. 7:**
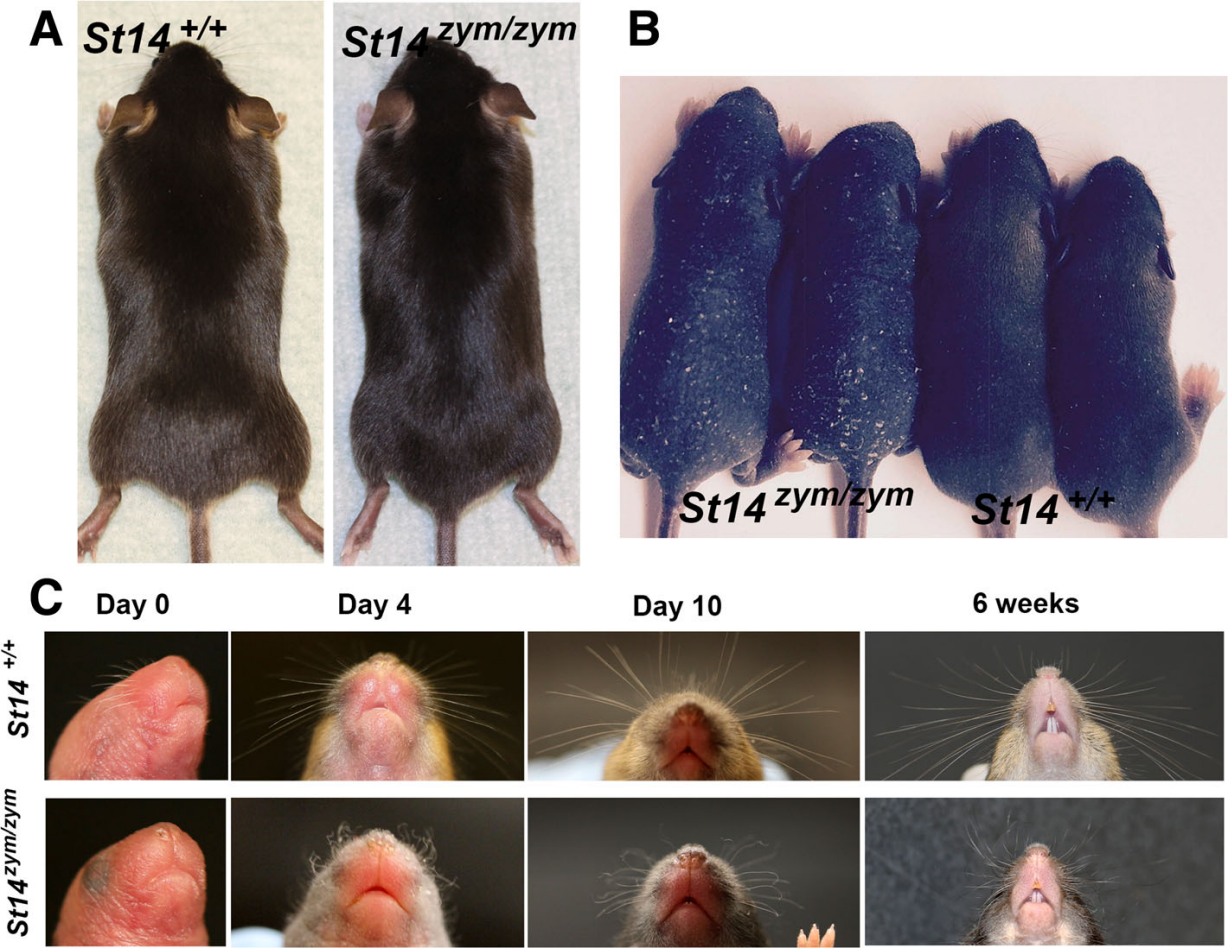
Matriptase zymogen conversion is required for normal whisker and pelage hair development. **a** Representative examples of the outward appearance of 1-month-old *St14* ^*+/+*^ mice (left) and *St14* ^*zym/zym*^ littermates (*right*). **b** Delayed pelage hair eruption in *St14* ^zym/zym^ mice. Representative example of two *St14* ^zym/zym^ pups and two wildtype littermates at day 6. **c** Loss of matriptase zymogen conversion causes whisker defects. Representative examples of whiskers from *St14* ^+/+^ (*top panels*) mice and *St14* ^zym/zym^ (*bottom panels*) littermates at birth (0 days), 4 days, 10 days, and 6 weeks. *St14* ^zym/zym^ mice display short, curly, and kinked whiskers

Matriptase is critical for development and maintenance of the hair follicle compartment of mice, as shown by congenital [6, 7] and conditional [9] matriptase ablation, respectively. The fur of adult *St14* ^zym/zym^ mice was unremarkable (Fig. 7a). However, pelage hair eruption was slightly delayed in *St14* ^zym/zym^ pups, as revealed through side-by-side comparison of *St14* ^zym/zym^ pups and wildtype littermates at days 4–6 (Fig. 7b). Likewise, whiskers, which are present in wildtype mice at birth, were absent in newborn *St14* ^zym/zym^ pups (Fig. 7c, Day 0). When they erupted later in postnatal development, the whiskers were often kinked and curly (Fig. 7c, Day 4, Day 10, and 6 weeks).

### Membrane-anchored prostasin stimulates PAR-2 activation by zymogen-locked matriptase

**Fig. 8:**
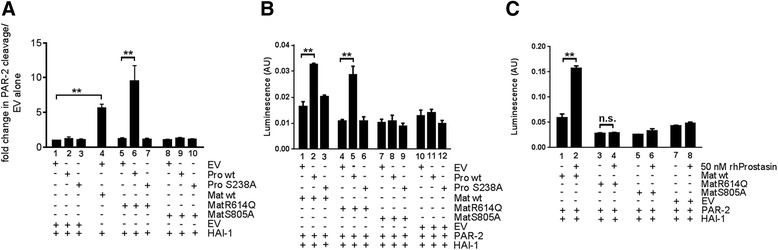
Matriptase zymogen is an efficient PAR-2 activator in the presence of membrane-anchored prostasin and HAI-1. **a** KOLF cells were transfected with plasmids encoding PAR-2-AP fusion protein and HAI-1 in combination with expression vectors for wildtype (lanes 2, 6, and 9) prostasin, catalytically inactive (S238A, lanes 3, 7, and 10) prostasin, wildtype (lane 4), zymogen-locked matriptase (lanes 5–7), or catalytically inactive matriptase (lanes 8–10). The corresponding empty vectors (EV) for prostasin (lanes 1, 4, 5, and 8) and matriptase (lanes 1–3) were used to ensure that transfections were performed with the same total amount of DNA. Data are shown as mean ± SD of duplicate transfections of free AP released to the media after 4 h standardized to the AP remaining on the cells. **P < 0.0001, one-way ANOVA. The data represent four independent experiments. **b** HEK293 cells were co-transfected with pSRE-firefly luciferase and pRL-Renilla luciferase reporter plasmids in combination with expression vectors for PAR-2 and HAI-1 (bars 1–12), wildtype (Mat wt) (bars 1–3), zymogen-locked (MatR614Q) matriptase (bars 4–6), catalytically inactive (MatS805A) matriptase (bars 7–9), or corresponding EV (bars 10–12) in combination with wildtype (WT) prostasin (bars 2, 5, 8, and 11), catalytically inactive (S238A) prostasin (bars 3, 6, 9, and 12), or corresponding EV (bars 1, 4, 7, and 10). Data are shown as mean ± SD of the mean of triplicate transfections of firefly luciferase light units/Renilla luciferase light units. **P < 0.0001, one-way ANOVA analysis. The data are representative of three similar experiments. **c** Zymogen-locked matriptase activity is dependent on membrane anchorage of prostasin. HEK293 cells were co-transfected with pSRE-firefly luciferase and pRL-Renilla luciferase reporter plasmids in combination with expression vectors for PAR-2 (bars 1–8), wildtype (Mat wt) matriptase (bars 1 and 2), zymogen-locked (MatR614Q) (bar 3 and 4) matriptase, catalytically inactive (MatS805A) matriptase (bars 5 and 6), or corresponding EV (bars 7 and 8) and human recombinant prostasin (bars 2, 4, 6, and 8) or vehicle (bars 1, 3, 5, and 7). Data are shown as the mean ± SD of triplicate transfections of firefly luciferase light units/Renilla luciferase light units. *P < 0.0001 and n.s. = not significant, one-way ANOVA. The data are representative of three similar experiments

To further investigate the ability of zymogen-locked matriptase to induce the cleavage of substrates, we used two previously established assays in which the activation of the established matriptase target substrate, PAR-2, is measured [46, 47]. PAR-2 is a G-protein-coupled receptor activated by the proteolytic removal of a short N-terminal peptide by trypsin-like serine proteases. Matriptase is a potent activator of PAR-2, and wildtype prostasin can enhance matriptase-dependent PAR-2 activation [46]. The first assay is performed in KOLF cells, which are PAR-1-deficient lung fibroblasts, and takes advantage of a PAR-2-AP fusion protein from which AP is released after PAR-2 N-terminal cleavage. In this way, matriptase activity can be quantified by the measurement of AP release to the medium (Fig. 8a). The second assay detects the amount of activated PAR-2 through the induction of transcription of a serum response element-luciferase reporter plasmid (Fig. 8b, c). Expression of wildtype prostasin or catalytically inactive prostasin (in which serine 238 of the catalytic serine-histidine-aspartate triad was mutated to alanine) in KOLF cells did not induce PAR-2 cleavage (Fig. 8a, compare bar 1 with bars 2 and 3). Expression of wildtype matriptase, but not zymogen-locked matriptase, induced PAR-2 cleavage in the absence of prostasin (Fig. 8a, compare bars 1, 4, and 5). Interestingly, however, zymogen-locked matriptase induced robust cleavage of PAR-2 when co-expressed with wildtype (Fig. 8a, compare bars 6 and 1), but not catalytically inactive (Fig. 8a, compare bars 7 and 1) prostasin. No increase in PAR-2 cleavage was observed when catalytically inactive matriptase was expressed alone (Fig. 8a, compare bars 1 and 8) or was co-expressed with wildtype or catalytically inactive prostasin (Fig. 8a, compare bar 1 with bars 9 and 10). Unlike PAR-2 cleavage in KOLF cells, neither wildtype matriptase nor zymogen-locked matriptase were able to induce significant PAR-2-dependent serum response element activity when expressed alone (Fig. 8b, compare bars 1 and 4 with bar 10). However, both wildtype and zymogen-locked matriptase induced serum response element activity when co-expressed with wildtype (Fig. 8b, compare bar 1 with bar 2 and bar 4 with bar 5), but not catalytically inactive prostasin (Fig. 8b, compare bar 1 with bar 3 and bar 4 with 6). The fold induction of PAR-2 activation was less robust in this assay, possibly reflecting different levels of endogenously expressed PAR-2-activating proteases in the two cell types and/or a high constitutive activity of the serum response element-luciferase reporter gene. Catalytically inactive matriptase was unable to induce serum response element activity, whether expressed alone (Fig. 8b, compare bars 7 and 10) or in the presence of wildtype (Fig. 8b, compare bars 8 and 11) or catalytically inactive (Fig. 8b, compare bars 9 and 12) prostasin. In the absence of co-expressed matriptase, neither wildtype (Fig. 8b, compare bars 10 and 11) nor catalytically inactive (Fig. 8b, compare bars 10 and 12) prostasin induced serum response element activity. Similar to wildtype prostasin expressed by transfection, exogenously added soluble prostasin stimulated serum response element activity induced by wildtype matriptase (Fig. 8c, compare bars 1 and 2). Interestingly, however, unlike transfected wildtype prostasin, soluble prostasin was unable to induce serum response element activity in the presence of zymogen-locked matriptase (Fig. 8c, compare bars 3 and 4). Catalytically inactive matriptase did not induce serum response element activity when expressed alone (Fig. 8c, compare bars 5 and 7) or in the presence of soluble prostasin (Fig. 8c, compare bars 6 and 8), and soluble prostasin by itself did not induce serum response element activity (Fig. 8c, compare bars 7 and 8). The ability of catalytically active prostasin, but not catalytically inactive prostasin, to promote zymogen-locked matriptase-dependent PAR-2 activation may indicate that prostasin, when activated by zymogen-locked matriptase, executes the activation site cleavage of PAR-2 or that the intrinsic activity of the matriptase zymogen is stimulated more effectively by catalytically active prostasin. Taken together, these data show that the matriptase zymogen can induce PAR-2 activation in the presence of prostasin, and that prostasin must be catalytically active and membrane-anchored to facilitate matriptase zymogen activation of PAR-2. The ability of catalytically active prostasin, but not catalytically inactive prostasin, to promote zymogen-locked matriptase-dependent PAR-2 activation may indicate that prostasin, when activated by zymogen-locked matriptase, executes the activation site cleavage of PAR-2 or that the intrinsic activity of the matriptase zymogen is stimulated more effectively by catalytically active prostasin.

## Discussion

The matriptase-prostasin system was originally proposed to be a simple cascade in which matriptase activated prostasin to execute the biologic functions of the system [42]. Indeed, phenotypic characterization of mice with congenital matriptase deficiency [6], conditional matriptase deficiency [9], matriptase hypomorphic mice [48], mice with congenital prostasin deficiency [43], conditional prostasin deficiency [49], prostasin hypomorphic mice and rats [50], mice expressing zymogen-locked endogenous prostasin [41], and mice expressing catalytically inactive prostasin [45] supports that this may indeed be the case in at least one organ – the hair follicle, which requires both the expression, zymogen conversion, and full activity of each of the two proteases for normal function. However, studies of these genetically modified animals and complementary cell-based studies have also revealed that the functional interactions between matriptase and prostasin, as well as their individual functions in other tissues, are much more complex and still incompletely understood. Thus, the simple linearity of the protease cascade was challenged by the ability of prostasin to efficiently activate matriptase on the surface of cultured cells [46], and by genetic epistasis analysis placing prostasin as either upstream [47] or downstream of matriptase [42, 48], depending on the specific context. More detailed cell-based analysis has partially reconciled these contradictory findings by showing that matriptase and prostasin are capable of forming a reciprocal zymogen activation complex stimulating the conversion of both the matriptase and the prostasin zymogens [37]. Even more unexpectedly, many biological functions of prostasin were recently shown to be independent of both prostasin zymogen conversion and prostasin catalytic activity [41, 45], although, paradoxically, even catalytically inactive prostasin requires strict developmental regulation by cognate serine protease inhibitors [43].

The current study uncovers additional complexity of the matriptase-prostasin system by showing that matriptase zymogen conversion is dispensable for key functions of the protease in epithelial development and function in mice. This capacity of a serine protease zymogen being able to execute essential proteolytic functions in vivo in the absence of zymogen conversion is rare, and to our knowledge, has only been unequivocally demonstrated for vampire bat plasminogen activator [51, 52]. In this regard, it is worthwhile noting that activated matriptase is rapidly inhibited by HAI-1 in epithelial cells, and the ratio of matriptase zymogen to activated, inhibitor-free matriptase is likely to be high [53]. It follows that the majority of available matriptase proteolytic activity on the cell surface may well originate from the zymogen, despite its lower rate of substrate catalysis. In support of this, data presented in this paper show that zymogen-locked matriptase, in the presence of HAI-1, which inhibits activated matriptase but not zymogen-locked matriptase, induces prostasin-dependent PAR-2 activation as efficiently as wildtype matriptase in a cell-based assay. Aligned with this, we found that zymogen-locked matriptase retains the ability to stimulate epidermal prostasin zymogen conversion in vivo, and that mice expressing zymogen-locked endogenous matriptase retain the ability to regenerate epidermis, a process recently shown to involve prostasin proteolytic activity [45]. Additional evidence for the engagement of the matriptase zymogen in cell surface proteolysis is provided by the curious observation that active site inhibitors of matriptase, including small molecule inhibitors and an antibody-based inhibitor, were either ineffective or needed concentrations of inhibitor that were multiple orders of magnitude higher than the K_i_ of the inhibitor for the activated matriptase serine protease domain to inhibit matriptase-dependent proteolysis on the cell surface [15, 54, 55].

It still needs to be shown how the current findings translate to human matriptase. Like rodent matriptase ([36] and the current study), the human matriptase zymogen is endowed with considerable enzymatic activity, as determined by the capacity of the soluble recombinant serine protease domain to auto-activate [34], the capacity of full length human matriptase zymogen to incorporate an activity-based probe in a cell-based assay [56], and the dependence of the matriptase catalytic triad for human matriptase to undergo activation site cleavage in cell-based assays [30, 35].

In the hair follicle and, likely, the interfollicular epidermis, matriptase and prostasin may co-localize constitutively on the plasma membrane [9, 57]. However, in most polarized epithelia and epithelial cells, matriptase is confined to the basolateral membrane and prostasin to the apical membrane [9, 10, 32, 58, 59], although prostasin appears to be required for matriptase zymogen conversion even in polarized epithelia [47, 60]. It has been proposed that matriptase and prostasin are brought together in polarized epithelia to facilitate mutual zymogen conversion only after disruption of homeostasis and loss of tight junction function [46]. While this may indeed represent a major pathway for activation of the matriptase-prostasin system during epithelial restoration [61], matriptase and prostasin are required not only for epithelial restoration, but also for normal homeostatic functions of many polarized epithelia [9, 10, 12, 62–64]. The current study, when combined with our recent studies of mice expressing zymogen-locked and catalytically inactive endogenous prostasin, now indicate that epithelial homeostasis may be maintained largely independent of conversion of either the matriptase zymogen or the prostasin zymogen. Alternatively, a polarized epithelium at homeostasis may display low levels of activated matriptase and prostasin due to the curious routing of prostasin to the basolateral membrane prior to its final delivery to the apical membrane [65].

## Conclusions

In summary, using gain and loss of function genetics in mice, we have found that the matriptase zymogen is a biologically active molecule that sustains epithelial development and homeostasis, and can cause pathology when mis-expressed. This finding advances our mechanistic understanding of matriptase and has important implications for the development of strategies for therapeutic targeting of this membrane-anchored protease in inflammatory, proliferative, and degenerative diseases.

## Methods

### Animal work

*St14* ^–/–^, *Prss8* ^–/–^, and *Prss8* ^zym/zym^ mice have been previously described [6, 41, 49]. All studies were littermate controlled to avoid genetic background differences from confounding data interpretation. Both males and females were used in the study.

### Generation of keratin-5-matriptase zymogen-locked and catalytically inactive transgenic mice

Keratin-5-matriptase transgenic mice were generated by performing site-directed mutagenesis on the full-length 3.1-kb mouse matriptase cDNA (GenBank AF042822) inserted into the pBK5-vector, which contains the 5.2-kb bovine keratin-5 regulatory sequences, β-globin intron 2, and 3′-polyadenylation sequences [14], by using the QuickChange Site Directed Mutagenesis Kit (Stratagene, La Jolla, CA). Site-directed mutagenesis primers for generating zymogen-locked matriptase, mutating the arginine 614 to glutamine, were F: 5′-CCTTTACCAAACAGGCTCAAGTGGTTGGTGGC-3′, R: 5′- GCCACCAACCACTTGAGCCTGTTTGGTAAAGG-3′. Primers for generating catalytically inactive matriptase, mutating the serine 805 to alanine, were F: 5′- TCCTGCCAGGGTGACGCTGGTGGCCCCTTG-3′, R: 5′- CAAGGGGCCACCAGCGTCACCCTGGCAGGA-3′. The linearized vectors were microinjected into the male pronucleus of FVB/NJ zygotes, which were implanted into pseudopregnant mice. Transgenic lines were established and maintained in an FVB/NJ background in the hemizygous state. The transgenic mice were genotyped by PCR amplification of genomic DNA extracted from tail biopsies using the following primers: 5′-CGTGCTGGTTATTGTGCTGTCT-3′ and 5′-GCTACCCATGGTTTTGGCGGTC-3′ [14], followed by direct sequencing of the amplified DNA.

### Generation of matriptase zymogen-locked knock-in mice

A custom ZFN was designed by Sigma Aldrich to cleave the murine *St14* gene encoding matriptase. The ZFN bound the *St14* gene 55 bp downstream from the desired point of mutation at the sequence 5′-GCCCTGGCAGGTGAGCCTCCacgccCTGGGCCAGGGCCACTTG-3′ (small letters indicate cleavage site). A 2000 bp DNA donor fragment identical to the genomic sequence of mouse *St14*, except for the desired di-nucleotide substitution, CGC to CAA, changing arginine 614 to glutamine, was purchased from Blue Heron Biotech (Bothell, WA) spanning 1000 bp upstream and 1000 bp downstream from the desired point mutation. The donor DNA also contained three synonymous base pair changes (shown in Fig. 2a) to minimize the cleavage of the donor DNA by the ZFN. The linearized donor DNA and ZFN mRNA were microinjected into the male pronucleus of FVB/NJ zygotes, which were implanted into pseudopregnant mice. The ensuing founders were screened for the introduction of the desired mutation using the primers WT F; 5′-CTTTACCAAACAGGCTCGC-3′, MUT F; 5′-CTTTACCAAACAGGCTCAA-3′, R WT + MUT 5′-CCATCACCCTGCTATAGTGC-3′. All founders positive for the insert were subsequently analyzed by direct sequencing by using primers located upstream of the DNA template and downstream of the di-nucleotide substitution to preclude PCR amplification of randomly inserted donor DNA. Three founders positive for the mutation were identified, two of which transmitted the edited *St14* allele when mated to Black Swiss mice. Offspring were genotyped by PCR using primers 5′-GCACACCCAGTGGAGATCAGA-3′ and 5′-CCAGCCAGTCAGGAGAGATGA-3′, which amplify a 250 bp fragment centered around the dinucleotide substitution. The CGC to CAA mutation results in the loss of a Cac8I restriction site (GCN˅NGC) and the simultaneous generation of a SmlI restriction site (C˅TYRA˄G). For genotyping, 5 μL of PCR product was digested overnight to completion with restriction enzymes Cac8I or SmlI.

### RT-PCR and quantitative real-time PCR analysis

Total skin RNA from newborn pups was homogenized using Precellys matrix beads and the Precellys 24 high-throughput tissue homogenizer (Bertin Technologies, Miami, FL) and then purified using the RNeasy mini kit (Qiagen, Hilden, Germany). cDNA was amplified by reverse transcription, followed by PCR amplification using the High Capacity cDNA Reverse Transcriptase Kit (Applied Biosystems, Foster City, CA), as recommended by the manufacturer. To specifically detect transgenic keratin-5-matriptase mRNA transcripts, PCR was performed on cDNA using the matriptase exon 1-specific primer R; 5′-GCTACCCATGGTTTTGGCGGTC-3′ and a primer complementary to exon 2 of the rabbit β-globin gene F; 5′-CACGTGGATCCTGAGAACTTCAG-3′. Quantitative real-time PCR analysis of matriptase was performed with SYBR Green PCR Master Mix (Applied Biosystems) according to the manufacturer’s instructions using the following primers: Total matriptase, 5′-AGATCTTTC TGGATGCGTATGAGA-3′ and 5′-GGACTTCATTGTACAGCAGCTTCA-3′; Transgenic matriptase, 5′-GCTACCCATGGTTTTGGCGGTC-3′ and 5′-CACGTGGATCCTGAGAACTTCAG-3′ (annealing temperature 60 °C, denaturation temperature 95 °C, extension temperature 72 °C, 40 cycles). Matriptase expression levels were normalized against S15 levels in each sample and amplified with the primers 5′-TTCCGCAAGTTCACCTACC-3′ and 5′-CGGGCCGGCCATGCTTTACG-3′.

### Protein extraction from mouse tissue

Tissues were dissected, snap frozen on dry ice, and stored at −80 °C until homogenization in ice-cold lysis buffer containing 1% Triton X-100, 0.5% sodium-deoxycholate in phosphate-buffered saline (PBS) plus Proteinase Inhibitor Mixture (Sigma) and incubation on ice for 10 min. The lysates were centrifuged at 20,000 × *g* for 20 min at 4 °C to remove the tissue debris, and the supernatant was used for further analysis. The protein concentration was measured with the BCA assay (Pierce).

### Western blot of skin from K5-matriptase transgenic mice

Samples were mixed with 4 × SDS sample buffer (NuPAGE, Invitrogen) containing 7% β-mercaptoethanol and boiled for 10 min. The proteins were separated on 4–12% BisTris NuPage gels and transferred to 0.2-μm pore size PVDF membranes (Invitrogen) blocked with 5% non-fat dry milk in Tris-buffered saline (TBS) containing 0.05% Tween 20 (TBS-T) for 1 h at room temperature. The PVDF membranes were probed with primary antibodies diluted in 1% non-fat dry milk in TBS-T overnight at 4 °C. Antibodies used for mouse lysates were sheep anti-human matriptase (catalogue no. AF3946, R&D Systems) and mouse anti-human prostasin (catalogue no. 612173, BD Transduction Laboratories, San Jose, CA). α-tubulin antibodies were used as a loading control (catalogue no. 9099S, Cell Signaling, Beverly, MA). The next day, the membranes were washed three times for 5 min each in TBS-T and incubated for 1 h with alkaline phosphatase-conjugated secondary antibodies (Thermo Scientific). After three 5-min washes with TBS-T, the signal was developed using nitro blue tetrazolium/5-bromo-4-chloro-3-indolyl phosphate solution (Pierce).

### Simple Western size separation of proteins from *St14* gene-edited mice

The Peggy Sue capillary electrophoresis system [40] (Protein Simple, Wallingford, CT, USA) was used to analyze matriptase and prostasin in lysates from newborn mice. Briefly, snap frozen skin, kidney, lung or intestines from newborn mice were lysed in T-PER lysis buffer with proteinase and phosphatase inhibitors (Pierce) as well as the serine protease inhibitor AEBSF to a final concentration of 0.5 mM (Sigma). The lysates were spun at 15,000 × *g* for 15 min at 4 °C and the supernatant was used for the analysis. A final concentration of 1 μg total protein per μL of sample was analyzed with the antibodies, sheep anti-human matriptase (catalogue no. 3946, R&D Systems), mouse-anti human prostasin (catalogue no. 612173, BD Transduction Laboratories), and β-actin as a loading control (catalogue no. ab6276-100, Abcam) according to the manufacturer’s instructions for size separation of proteins. Quantitative analysis of immunoreactive proteins was performed using the Compass Software, which automatically measures the luminescence signal as a function of the amount of protein present in the sample at a certain molecular mass. The software calculates the area under the curve as a measure of the amount of protein [40].

### Immunohistochemistry

Organs were collected from mice and fixed in 4% paraformaldehyde in PBS for 24 h, embedded into paraffin, and sectioned. Tissue sections were cleared with xylene-substitute (Safe Clear, Fisher Scientific), rehydrated in a graded series of alcohols, and boiled in Reduced pH Retrieval Buffer (Bethyl, Montgomery, TX) for 20 min for antigen retrieval. The sections were blocked for 1 h in PBS containing 10% horse serum and incubated at 4 °C overnight with sheep anti-human matriptase (catalogue no. AF3946, R&D Systems). The slides were washed three times in PBS and incubated at room temperature for 45 min with secondary antibodies, using Alexa Fluor 594-labeled donkey anti-sheep (Invitrogen) or a biotin-conjugated anti-mouse secondary antibody (1:1000, Vector Laboratories, Burlingame, CA) and a Vectastain ABC kit (Vector Laboratories) using 3,3-diaminobenzidine as the substrate (Sigma-Aldrich). The tissue sections were washed three times for 5 min with PBS and mounted with VectaShield Hard Set Mounting Medium (Vector Laboratories).

### Measurement of stratum corneum and epidermal thickness

Epidermal thickness (excluding stratum corneum) and stratum corneum thickness were measured using the ScanScope software from Aperio. Measurements were made in the same 0.5–1 cm segment of dorsal skin and were calculated by averaging independent measurements per 100 μm skin specimen*.*

### Transepidermal fluid loss assay

Transepidermal fluid loss assay was performed exactly as previously described [6, 7].

### Intestinal epithelial permeability assay

A 22 mg/mL fluorescein isothiocyanate (FITC)-labeled dextran (average 4000 g/mol, Sigma) in PBS was instilled directly into the stomach of mice at a volume of 10 μL per g of body weight. Intillations were made using a 1.5-cm long, bulb-tipped gastric gavage needle (Roboz, Gaithersburg, MD) attached to a 1 mL syringe. After 3 h, blood was collected from the heart of freshly euthanized mice and clotted for 2 h at room temperature. Sera were then isolated via centrifugation at 800 × *g* for 10 min at room temperature, upon which the clear upper phase was transferred into a new tube. All samples were immediately stored at –80 °C until analysis. For the analysis, 50 μL of each sample was diluted 1:3 in PBS (Gibco-Invitrogen, Carlsbad, CA) and dispensed into a 96-well plate. The concentration of FITC-dextran was determined by reading the fluorescence at 535 nm after excitation at 485 nm using a Victor3V spectrophotometer (PerkinElmer, Waltham, MA).

### Cutaneous wound repair

Full thickness incisional skin wounds (15 mm) were made in the interscapular dorsum. Healing was assessed by daily inspection of wounds by an investigator blinded to genotype, as described previously [66]. Macroscopic closure of the incisional interface was evaluated both visually and by palpation, and the wound was scored as healed when only a minimal residual defect, which resolved very slowly with time, was observed.

### Cell culture and PAR-2 activation assay

HEK293 cells (ATCC, Manassas, VA) were grown in DMEM supplemented with 2 mM L-glutamine, 10% fetal bovine serum, 100 units/mL penicillin, and 100 μg/mL streptomycin (Invitrogen) at 37 °C in an atmosphere of 5% CO_2_. Cells (250,000/well) were plated in 24-well poly-L-lysine-coated plates and grown for 24 h. Cells were co-transfected with pSRE-firefly luciferase (50 ng), pRL-*Renilla* luciferase (20 ng), pcDNA3.1 containing a full-length human protease-activated receptor (PAR)-2 cDNA (100 ng) (Missouri S&T cDNA Resource Center), pcDNA3.1 containing human matriptase (100 ng), matriptase locked in its zymogen form (R614Q) or catalytically inactive matriptase (S805A) [37], pcDNA3.1 containing human HAI-1 (100 ng), pIRES2-EGFP human prostasin (100 ng), or catalytically inactive prostasin (S238A) [37] as indicated in the individual experiments. Lipofectamine (Invitrogen) was used as the transfection agent according to the manufacturer’s instructions. The pSRE-firefly luciferase is a luciferase reporter gene under the control of a multimerized serum-response element. The transfection medium was changed at 18 and 48 h after transfection, and the cells were serum-starved overnight. Cells were lysed and luciferase activity was determined using the Dual-Luciferase assay kit (Promega) according to the manufacturer’s instructions. Luminescence was measured using a Wallac Victor2 1420 multilabel counter (PerkinElmer Life Sciences), and the serum-response element activity was determined as the ratio of firefly luciferase to *Renilla* luciferase light units.

### Cleavage assay of PAR fusion proteins

PAR-1 knockout (*F2r* ^*–/–*^) lung fibroblasts (KOLF) [67] were grown in DMEM supplemented with antibiotics and 5% fetal bovine serum. Transfections were performed with the TransitLT1 transfection reagent (Euromedex) in complete medium. The cells were transfected with pcDNA3.1SEAP-PAR2 encoding a secreted AP-fused PAR-2 protein [24], full-length human matriptase cDNA in the pcDNA3.1 vector, or the mutants matriptase R614Q and matriptase S805A, in combination with full-length human prostasin cDNA or the catalytically inactive mutant prostasin S238A. For all transfections, pcDNA3.1HAI-1 was included to prohibit the constitutive activation of PAR-2 activation. Cells were used for experiments 48 h after transfection to determine the cleavage of the co-transfected substrate SEAP-PAR2. After washing the monolayer for 1 h with OptiMEM, cells were incubated for 4 h with vehicle only and medium was collected (fraction 1). Trypsin (10 nM for 20 min) was then used to release all surface exposed SEAP moieties (fraction 2). SEAP hydrolysis of para-nitrophenyl phosphate in fractions 1 and 2 was assessed at OD405. The ratio between SEAP hydrolysis in 4 h (fraction 1) and a trypsin strip (fraction 2) was determined for all conditions. It was then normalized to the OD405 ratio of cells co-transfected with the SEAP protein and empty vector.

### Statistics and general methods

All analyses were performed and graphs generated using the Prism5 software (GraphPad Software Inc.) For comparison of two samples, Student’s *t* test was used. For multiple comparisons, one-way ANOVA was used with adjustment for multiple comparisons (non-parametric for the gavage study). Transepidermal fluid loss rates were analyzed by linear regression and wound healing by the log-rank test. All tests were two-tailed. Exact sample sizes (biological replicates) are indicated for each experiment.

#### Choice of sample size

Figure 1c–f: Large litters containing similar numbers of wildtype and transgenic mice were chosen for analysis. Replicated two times. Figure 2k: Sample size based on tissue available for analysis; performed one time. Figures 5b and i, 6i and j: Sample size based on number of litters available for analysis; performed one time. Figure 8a–c: Sample size based on variation observed in prior use of assays [24, 37]; replicated three times.

#### Inclusion/exclusion criteria

Littermates of transgenic mice and wildtype controls were included. Mice were not excluded from the study.

#### Randomization of study

The study compared mice of different genotypes and cells transfected with different plasmids. Randomization is not applicable.

#### Blinding

Blinded as to genotype in Figs. 1k, 4a and i, 6i and j, 7b and c.

#### Justification of statistical test

Parametric tests were used when normal distribution and equal variance could be assumed. Non-parametric tests had sample sizes of more than 5.

## Additional file

Additional file 1: Raw supporting data. (XLSX 5530 kb)

## Abbreviations

AP: alkaline phosphatase
FITC: fluorescein isothiocyanate
HAI-1: hepatocyte growth factor activator inhibitor
PAR-2: proteinase activated receptor-2
ZFN: zinc finger nuclease
